# Unmet need for COVID-19 vaccination coverage in Kenya

**DOI:** 10.1016/j.vaccine.2022.02.035

**Published:** 2022-03-18

**Authors:** Samuel K. Muchiri, Rose Muthee, Hellen Kiarie, Joseph Sitienei, Ambrose Agweyu, Peter M. Atkinson, C. Edson Utazi, Andrew J. Tatem, Victor A. Alegana

**Affiliations:** a Population Health Unit, Kenya Medical Research Institute-Wellcome Trust Research Programme, Nairobi, Kenya; b Department of Health Informatics, Monitoring and Evaluation, Ministry of Health, Nairobi, Kenya; c Epidemiology and Demography Department, KEMRI-Wellcome Trust Research Programme Nairobi, Kenya; d Lancaster Environment Centre, Lancaster University, Lancaster LA1 4YQ, UK; e Geography and Environmental Science, University of Southampton, Highfield, Southampton SO17 1BJ, UK; f Institute of Geographic Sciences and Natural Resource Research, Chinese Academy of Sciences, Beijing 100101, China; g WorldPop, School of Geography and Environmental Science, University of Southampton, Southampton, UK; h Southampton Statistical Sciences Research Institute, University of Southampton, Southampton, UK

**Keywords:** COVID-19, Vaccination coverage, Bayesian conditional autoregressive, Spatial inequalities, CAR, Conditional Auto-regressive, COVAX, Coronavirus Disease of 2019 Vaccine Global Access facility, COVID-19, Coronavirus Disease of 2019, CPO, Condition Predictive Ordinate, CRA, Commission on Resource Allocation, DEM, Digital Elevation Model, DIC, Deviance Information Criterion, DTP, Diptheria-tetanus-pertussis, EUA, Emergency Use Authorization, FBO, Faith-Based Organization, GIS, Geographic Information System, INLA, Integrated Nested Laplace Approximation, KDHS, Kenya Demographic and Health Survey, KEPH, Kenya Essential Package for Health, MAE, Mean Absolute Error, MoH, Ministry of Health, NGO, Non-Governmental Organization, NPI, Non-Pharmaceutical Intervention, PBB, Pharmacy and Poisons Board, PIT, Probability Integral Transform, RCMRD, Regional Centre for Mapping of Resources for Development, RMSE, Root Mean Square Error, SAE, Small Area Estimation, SARS CoV-2, Severe Acute Respiratory Syndrome Coronavirus 2, WAIC, Watanabe-Akaike Information Criterion

## Abstract

COVID-19 has impacted the health and livelihoods of billions of people since it emerged in 2019. Vaccination for COVID-19 is a critical intervention that is being rolled out globally to end the pandemic. Understanding the spatial inequalities in vaccination coverage and access to vaccination centres is important for planning this intervention nationally. Here, COVID-19 vaccination data, representing the number of people given at least one dose of vaccine, a list of the approved vaccination sites, population data and ancillary GIS data were used to assess vaccination coverage, using Kenya as an example. Firstly, physical access was modelled using travel time to estimate the proportion of population within 1 hour of a vaccination site. Secondly, a Bayesian conditional autoregressive (CAR) model was used to estimate the COVID-19 vaccination coverage and the same framework used to forecast coverage rates for the first quarter of 2022. Nationally, the average travel time to a designated COVID-19 vaccination site (*n* = 622) was 75.5 min (Range: 62.9 – 94.5 min) and over 87% of the population >18 years reside within 1 hour to a vaccination site. The COVID-19 vaccination coverage in December 2021 was 16.70% (95% CI: 16.66 – 16.74) – 4.4 million people and was forecasted to be 30.75% (95% CI: 25.04 – 36.96) – 8.1 million people by the end of March 2022. Approximately 21 million adults were still unvaccinated in December 2021 and, in the absence of accelerated vaccine uptake, over 17.2 million adults may not be vaccinated by end March 2022 nationally. Our results highlight geographic inequalities at sub-national level and are important in targeting and improving vaccination coverage in hard-to-reach populations. Similar mapping efforts could help other countries identify and increase vaccination coverage for such populations.

## Highlights

• COVID-19 vaccination coverage was modelled using vaccination data from the Kenya Ministry of Health.
• The average travel time to a designated COVID-19 vaccination site was a key predictor of COVID-19 vaccination coverage.
• Bayesian modelling suggests inequalities in population vaccination coverage for COVID-19 at the sub-national level in Kenya.
• Vaccination coverage mapping can be a useful tool for targeting interventions.

## 1 Introduction

The World Health Organization (WHO) declared the coronavirus disease (COVID-19) a pandemic on 11^th^ March 2020 [1], with the first case in Kenya confirmed on 12^th^ March 2020 [2]. Since then, 295,028 cases and 5,378 fatalities had been reported by 31^st^ December 2021 [2], [3]. Kenya has experienced four COVID-19 epidemic waves since March 2020, with a fifth underway at the time of preparation of this manuscript [3]. Various non-pharmaceutical interventions (NPIs) have been implemented at different timepoints to slow down the spread of the virus so that health systems can cope with demand for illness management. These include mandatory wearing of masks in public places, a dawn-to-dusk curfew, physical distancing guidelines, closure of bars, restaurants and places of worship, restriction of movement in and out of counties with high infection rates, closure of schools and institutions and a ban on social gathering and meetings [4], [5]. Various pharmaceutical interventions have been trialed for treatment of COVID-19, and several are under investigation. These include anti-viral treatments, corticosteroids, and biological therapeutics [6], [7], [8], [9], [10], [11], [12], [13]. However, for sustainable control of the COVID-19 pandemic, vaccinations are required to be provided to all sections of the population [14], [15]. Knowledge of vaccination coverage gaps at national and sub-national levels is, thus, important for planning vaccination campaigns, targeting hard-to-reach populations and increasing access to vaccines in marginalized areas.

There are eight vaccines approved so far for emergency use by WHO [16], five of which are available in Kenya as of December 2021 [2], [17]. Their efficacies range from 66.7% to 95% [18], [19] with all demonstrating high degrees of protection from severe disease or death [20]. Kenya is one of the countries globally that is eligible for subsidized access to vaccines through the COVID-19 Vaccines Global Access facility (COVAX) [21]. The Government of Kenya has targeted to vaccinate 10 million people (38% coverage) aged 18 years and above by December 2021 [22], and 26 million people (99% coverage) by December 2022 [17].

Understanding geographical access to COVID-19 vaccination sites and coverage is important for effective planning of vaccination programmes. Beyond geographical access, other factors that may affect COVID-19 vaccination coverage at population level including vaccine availability and, individual factors such as literacy, vaccination perception and acceptability, and, household level factors including location (urban or rural) [23], [24], [25], [26]. To model the spatial inequalities in COVID-19 vaccination coverage, statistical approaches can be used which incorporate other geographic data. Such approaches have been applied previously in childhood vaccination for measles [27], [28], [29], [30], [31] and diphtheria-tetanus-pertussis (DTP) [27], [31], [32] vaccines. The use of small area estimation (SAE) methods [33], [34], [35], combined with population data [36], can estimate vaccination coverage at sub-national level to compare with national set targets as well as forecast these coverage estimates and associated uncertainties [37], [38], [39]. SAE and spatial statistical methods have been used in similar contexts to model malaria incidence in northern Namibia [40], lung cancer risk in Pennsylvania, the United States [41] and measles and DTP vaccination coverage in Afghanistan and Pakistan [29].

Here, the main objective was to estimate current (first 9 months) and future (forecast to March 2022 - the following 3 months) COVID-19 vaccination coverages at national and sub-national levels in Kenya since the start of the vaccination campaign in March 2021. This was undertaken using data assembled at the sub-national level on vaccination sites, number of people vaccinated and the eligible population over the age of 18 years. A secondary objective was to assess geographic accessibility to COVID-19 vaccination sites and evaluate the association between travel time, rurality, and age with vaccination coverage.

## 2 Methods

### 2.1 COVID-19 data

Vaccination data were assembled from the Kenya Ministry of Health (MoH) daily vaccination bulletins [2]. These data comprised the number of vaccinations primarily targeting priority groups representing health workers, security personnel, teachers, and vulnerable populations over 58 years [42], [43]. In Kenya, the first dose of the Oxford-AstraZeneca and the Sputnik V vaccines were administered from March 2021 and from September 2021, the Johnson and Johnson, Pfizer, Sinopharm and Moderna vaccines were included. Dose 1, therefore, represents the total number of doses administered for these five vaccines. These vaccines were issued Emergency Use Authorization (EUA) by the Kenya Pharmacy and Poisons Board (PBB). The administration of the second dose started in late May, and data for both the first and second doses were compiled separately. The daily bulletins are aggregated weekly at county level (Administrative level 1). However, it was not possible to identify individual adult age ranges vaccinated across all the priority groups. Data on the receipt of the first dose vaccinations were available from 6^th^ April 2021 and was unavailable from 13^th^ July 2021 onwards. On the other hand, data on the number of dose 2 vaccine administration were available from 9^th^ June onwards. Due to limitations in dose 1 data for the analysis, dose 1 and dose 2 data were combined to obtain “any dose” data that was used for analysis.

COVID-19 stringency index data were obtained from Our World in Data website [44]. The stringency index represents the level of strictness of the lockdown measures. The index was calculated using nine NPIs which include school and workplace closures, cancellation of public events, restrictions on public gatherings, closures of public transport, stay-at-home requirements, public information campaigns, restrictions on internal movements, and international travel controls. A detailed methodology for constructing the stringency index is provided elsewhere [45]. The national weekly rolling average COVID-19 case number data were obtained from the Our World in Data website [44].

### 2.2 COVID-19 vaccination sites

The list of approved COVID-19 vaccination sites was downloaded from the Ministry of Health website [2]. This represented 622 health facilities comprising dispensaries (*n* = 12), health centres (*n* = 55) and hospitals (county hospitals (*n* = 533), county referral hospitals (*n* = 15) and national referral hospitals (*n* = 7). The vaccination sites were further coded by ownership (Public and military, private, Faith-Based Organization (FBO) and Non-Government Organization (NGO)). Lower-level health facilities (dispensaries and health centres) have lower capacity to provide inpatient care for COVID-19 infections but have the capability to vaccinate due to the availability of cold storage facilities principally used for childhood immunisation programmes. These health facilities are part of a previously established spatial database of health facilities [46].

### 2.3 Population estimates for over 18 years

Fine spatial resolution population data was obtained from WorldPop [47]. WorldPop provides population estimates adjusted to match the official UN population estimates for 2020, and this was projected to 2021 using the UN medium variation national growth rate [48]. The methodology for modelling population is described elsewhere [49] and combines data from various population and housing censuses, human settlements, and covariates related to population distribution via machine learning approaches to generate a gridded prediction of population density at 100 m spatial resolution [50], [51]. The population raster data was then resampled to 1 × 1 km. It was necessary to define a suitable denominator population based on the age range of >18 years representing the priority groups targeted for vaccination (health workers, teachers, government officials, security forces, and individuals aged >58 years). The denominator population representing the target groups was computed as a product of the total population and modelled age proportion at the same spatial scale (1 km by 1 km). To estimate fine spatial resolution proportion of population >18 years, independent nationally representative household survey data with household census were used. The Kenya Demographic and Health Survey (KDHS) data available dates back seven years [52] and plans to conduct a national survey in 2020 were postponed. However, a new national DHS survey is currently ongoing which is estimated to be completed in 2022 [53]. The prediction of age proportions for >18 years used DHS cluster level data adjusting for rural and urban residence. Continuous age data was assumed to follow a Gamma distribution. These parameters of the Gamma distribution extracted at cluster level were interpolated spatially, and Monte-Carlo simulations were performed to draw age distributions at 1 km spatial scale. Further details of age distribution modelling are presented in the supplementary information.

### 2.4 Modelling travel time to COVID-19 vaccination centres

Geographic information system (GIS) data were assembled for estimating travel time to COVID-19 vaccination centres. These included roads assembled from the ministry of transport and updated via OpenStreetMap and Google Map Maker as detailed elsewhere [54], [55], rivers and lakes [56], [57], [58], national parks and reserves [56], [57], [58], Copernicus Sentinel-2 landcover at 20 m × 20 m spatial resolution, and digital elevation model (DEM) data at 30 m spatial resolution available from the Regional Centre for Mapping of Resources for Development (RCMRD) GeoPortal [59]. The methodology for estimating theoretical travel times to vaccination sites has been demonstrated elsewhere [54], [55], [60], [61], [62], [63] and was adopted in this manuscript. In brief, a cost distance algorithm based on walking (along footpaths or landcover classified as bare and agricultural) and motorized travel time (along major roads) was used. The travel speeds adopted were based on previous studies [54], [55], [62]. A correction for slope uphill or downhill was applied based on Tobler’s formulation [64]. The analysis was conducted at 1 × 1 km spatial resolution to produce a gridded surface of travel time to vaccination centres from any population location. Finally, the modelled travel time surface was combined with population density to produce a population-weighted travel time surface which excludes areas with no population.

### 2.5 Modelling COVID-19 vaccination coverage at sub-national level

To model the COVID-19 vaccination coverage rates, vaccination data were combined with a set of covariates to inform our prediction model. These covariates were chosen based on accessibility to vaccines and priority groups chosen for vaccination. The covariates used include the mean travel times to COVID-19 vaccination sites, proportion of the population in urban/rural areas and proportion of the adult population under 58 years/over 58 years. The mean travel time covariate used was categorical and was classified into four classes: 1 (within 1 hr), 2 (1–2 hrs), 3 (2–3 hrs) and 4 (beyond 3hrs), while the remaining covariates were continuous. For continuous covariates, the extracted values were the mean values at sub-national level, while for the categorical covariate, the extracted values were the majority classified values within that sub-national boundary. The covariates were then standardized and matched with the COVID-19 vaccination data at the county level.

The numerator for the coverage rates comprised the total number of vaccines administered (Y) while the denominator was the total target population (P). To model vaccination coverage across counties, a discrete spatial binomial regression model was implemented in a Bayesian framework using the Integrated Nested Laplace Approximations in R (R-INLA) [65], [66].

Let t(t=1,...,T) represent time in weeks, m(m=1,⋯.12) represent time in months and i=1,⋯,N represent the sub-national areas/counties. Denoting $Y_{t,i}$ as the number of vaccines administered in area i at time t, the model is given by:

$$Y_{t,i}\;\sim \;\;\mathrm{Binomial}\left(\mu_{t,i}\right)$$

$$\log\left(\mu_{t,i}\right)=\log\left(P_{t,i}\right)+\theta_{t,i}=\log\left(P_{t,i}\right)+X_{t,i}\beta +\alpha_{t(m)}+\omega_{i},$$

where $P_{t,i}$ is the population count corresponding to $Y_{t,i}\left(t\right)$, $X_{t,i}$ represents a vector of covariates and β, the corresponding regression coefficients. $\alpha_{t(m)}$ is a monthly temporal random effect modelled as a first-order autoregressive process, that is, $\alpha_{t(m)}\;N\left(\rho \alpha_{t(m)-1},1/\tau \right)$, where ρ is an autoregressive parameter and τ is a precision parameter. $\omega_{i}$ is a spatial random effect modelled as $\omega_{i}∼N\left(0,1/\tau_{\omega }\right)$, which was used to capture random spatial variation between the counties. Further, $\theta_{t,i}$=$X_{t,i}\beta +\alpha_{t(m)}+\omega_{i}$ is the log rate of vaccination (i.e. log of the average number of individuals vaccinated divided by the target population) for area i at time t. The Bayesian specification was completed by assigning zero-mean Gaussian prior distributions to the regression coefficients, the penalised complexity (PC) priors to the $AR\left(1\right)$ parameters [67], [68], and using a non-informative log-gamma prior on the precision parameter of the county-level random effect.

Model selection was conducted by comparing two models: Model 1 with covariates and temporal adjustments, but no spatial random effect and Model 2, which included the spatial random effect. Sensitivity analysis was conducted to select the best model. Model goodness of fit was assessed using deviance information criterion (DIC), Watanabe-Akaike information criterion (WAIC) and other INLA internal model evaluation metrics; the conditional predictive ordinate (CPO) and the probability integral transform (PIT) [69], [70]. The model predicted the vaccination coverage rates (posterior means) for the first nine months (i.e. April to December 2021) with 95% credible intervals, and, this was used as the basis for forecasting the coverage rates to the next three months (i.e. from January 2022 to March 2022).

For validation, cross-validation techniques were used to evaluate the predictive performance of the model. This was based on a 20% sub-set of data selected randomly and was used to compute the root mean square error (RMSE) which assesses the overall performance and accuracy of the model, the mean absolute error (MAE) which assesses the model bias, and the Pearson’s correlation co-efficient which evaluates the association between predicted and observed values. The closer the RMSE value is to zero, the better the prediction.

## 3 Results

### 3.1 Summary of the COVID-19 vaccination sites.

**Fig. 1:**
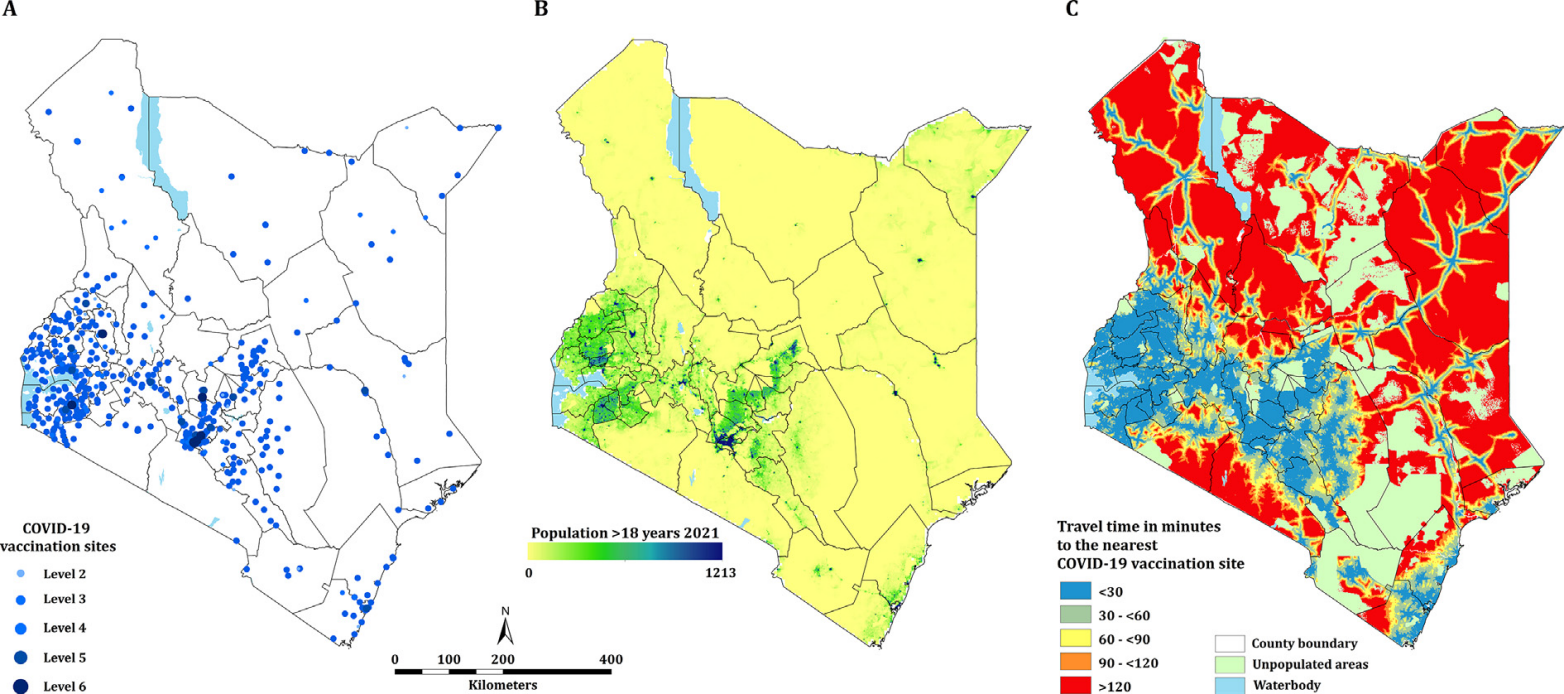
(**A**) Distribution of the approved vaccination sites categorized according to their KEPH levels (*n* = 622). (**B**) Population density distribution for >18 years per 1 km^2^. (**C**) Spatial accessibility to the COVID-19 vaccination sites.

Fig. 1A shows the spatial distribution of the 622 COVID-19 vaccination sites categorized according to the Kenya Essential Package for Health (KEPH) levels. In terms of ownership, 448 (72.0%) were public and 173 (27.8%) were private facilities. Majority of the COVID-19 vaccination sites are in areas of high population densities (Fig. 1A and B). Most of these vaccination sites were located around the Lake Victoria basin and the western, central, and coastal regions of Kenya. The Northern part of the country has a few scattered vaccination sites (Fig. 1**B**). The number of vaccination sites per county ranged from one in Bomet, Samburu and Elgeyo-Marakwet to 64 in Nairobi. With an average of 13 vaccination sites per county, 17 counties contained more than the average number nationally and accounted for 47.3% of the denominator population. In contrast, the arid counties which include Garissa, Isiolo, Mandera, Marsabit, Samburu, Tana River, Turkana and Wajir [71] hosted a total of 53 (8.5%) vaccination sites and accounted for 13.7% of the denominator population (Table SI 2).

### 3.2 Travel time to COVID-19 vaccination sites

**Fig. 2:**
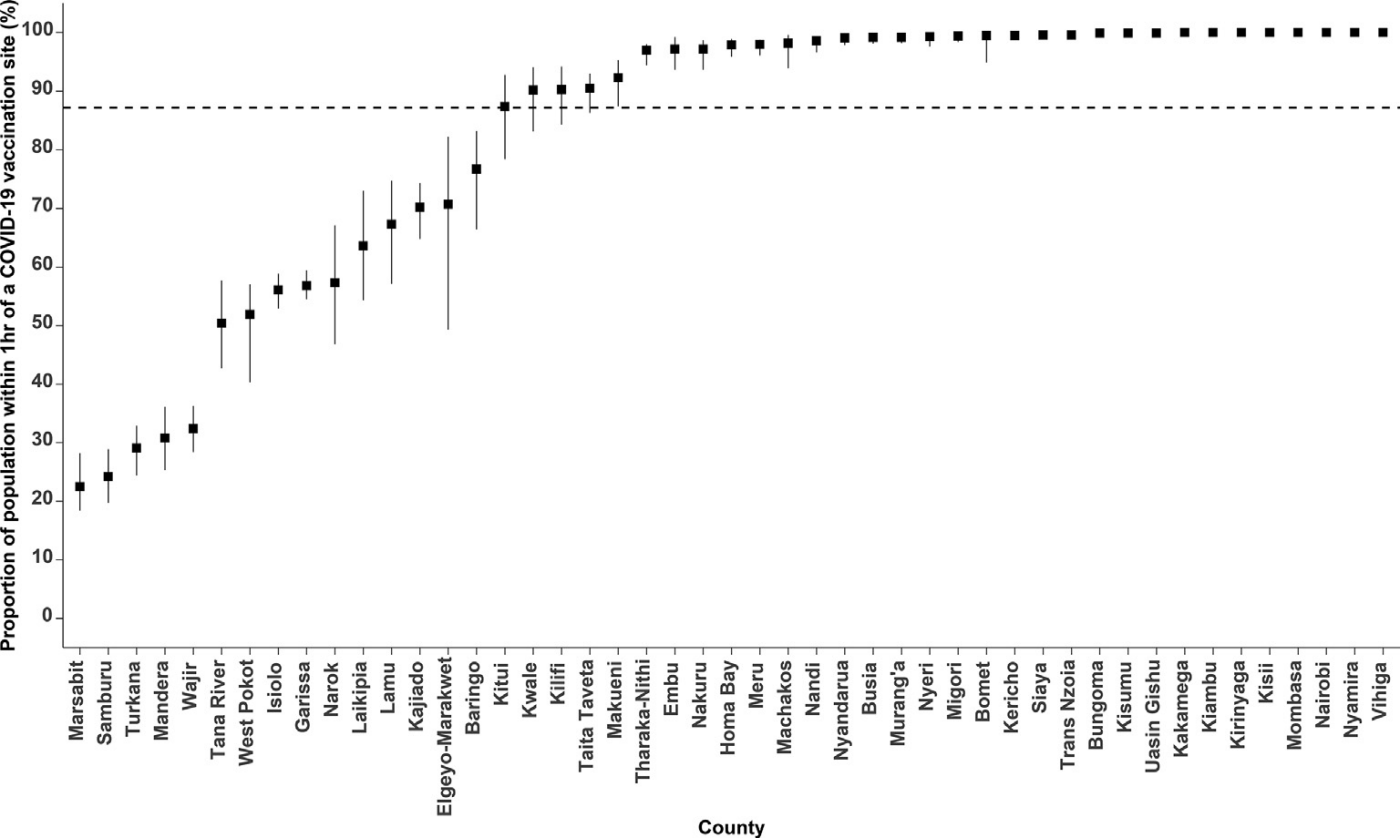
Proportion of population living within 1hr travel time to a COVID-19 vaccination site. The error bars represent the uncertainty intervals, derived by varying the mean speeds by ±20%. The dotted line represents the national average percentage of population living within 1 hr to a COVID-19 vaccination site*.*

Spatial access to vaccination sites varied across each of the 47 counties and was highly heterogeneous (Fig. 1C). Overall, the mean travel time to the nearest vaccination site was 75.5 min (Range: 62.9–94.5 min), which ranged from 5.9 min (Range: 4.8–7.4 min) in Nairobi to 294.0 min (Range: 244.9–367.7 min) in Marsabit counties. 11 (23.4%) counties had a mean travel time of over 2 hrs which include Garissa, Isiolo, Kajiado, Lamu, Mandera, Marsabit, Samburu, Tana River, Turkana, Wajir and West Pokot. These counties were the most marginalized with average travel times ranging from 137.3 min to 294.0 min. In Kenya, approximately 22.8 million of the adult population or 87.2% (Range: 84.4–89.0) reside within 1 hr to a COVID-19 vaccination site ranging from 22.5% (Range: 18.4– 28.2) in Marsabit county to 100% in Kakamega, Kiambu, Kirinyaga, Kisii, Mombasa, Nairobi, Nyamira and Vihiga counties. Five counties had less than 50% of their adult population within 1 hr of a vaccination site: Mandera, Marsabit, Samburu, Turkana and Wajir. It is notable that several large counties such as Turkana, Wajir, Tana River, West Pokot, Marsabit, Mandera, Isiolo and Garissa have very poor access as compared to several smaller counties such as Nairobi, Vihiga, Mombasa and Nyamira with all their population residing within 1hr of a vaccination site. However, exceptions were observed in large counties such as Nakuru, Makueni, Meru and Machakos with over 90% of their adult population within 1hr of a vaccination site. Fifteen (31.9%) counties were below the national average of proportion of population within 1hr of a COVID-19 vaccination site (Fig. 2).

### 3.3 Model results and validation

Table SI 1 compares the two models based on their DIC, WAIC and marginal log-likelihood. For any-dose vaccination coverage modelling, Model 2 performed better as indicated by the lower DIC and WAIC values and hence was selected for the subsequent modelling and forecasting of COVID-19 vaccination coverages. For model 2, the spatial random effect had a small variation at the county level of 3.703 (95% CI: 2.344–5.465) (Table 1). The model prediction performance was assessed based on the 20% sub-set data. The RMSE value was 1.123 and MAE value of 0.700. The Pearson’s correlation co-efficient was 0.965 indicating a very strong linear relationship between the observed and the predicted vaccination coverages. The scatter plot is shown in Fig. SI 1 (supplementary information).

**Table 1:** Estimates of the parameters of the fitted model for COVID-19 vaccination coverages. Reported are the posterior means and the 95% credible intervals (CI) of the exponentiated regression coefficients and other parameters. The categories - travel time within 1 hr, percentage population in urban areas and percentage population under 58 years - were the reference parameters for mean travel time, Rural/Urban and age covariates, respectively.

**Parameter**	**Mean**	**Credible Intervals (5%, 95%)**
Intercept	0.015	(0.010, 0.021)
Week	1.070	(1.070, 1.070)
**Mean travel time**		
<1 hr	1.000	–
1–2 hrs	0.935	(0.586, 1.417)
2–3 hrs	0.502	(0.274, 0.845)
>3 hrs	0.319	(0.180, 0.526)
**Rural/Urban**		
% of population in Urban	1.000	–
% of population in Rural	0.722	(0.616, 0.842)
**Age**		
% of population under 58 years	1.000	–
% of population over 58 years	1.376	(1.135, 1.652)
**Random effects (Hyperparameters)**		
Precision for Month (τ)	38.441	(9.298, 87.099)
Autocorrelation parameter for Month (ρ)	0.742	(0.461, 0.806)
Precision for County ($\tau_{\omega }$)	3.703	(2.344, 5.465)

Table 1 shows the posterior means and the 95% credible intervals of the fitted model parameters for the fixed effects and random effects (hyperparameters) respectively. For any dose vaccination coverage, there was generally an increase in the vaccination coverage through the weeks in that a 7.0% increase in vaccination coverage was observed. The probability of being vaccinated generally decreased with increase in mean travel times to the COVID-19 vaccination sites. For example, there is a 68.1% decrease in vaccination coverage for people residing in areas over 3 h travel time from a site. There was a negative association between the vaccination coverage and the proportion of population residing in rural areas with a 27.8% decline. There was a 37.6% increase in vaccination coverage among the population over 58 years.

### 3.4 Predicting the vaccination coverage rates at sub-national level

**Fig. 3:**
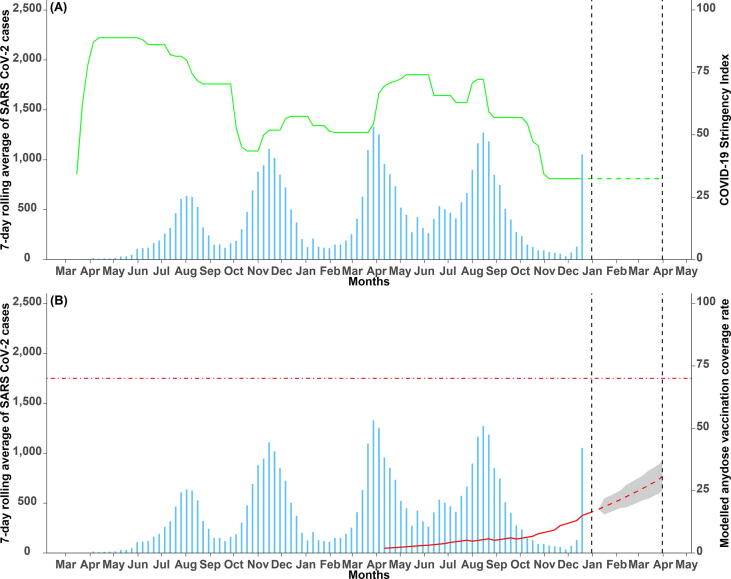
Weekly average SARS CoV-2 cases superimposed with (**A**) COVID-19 stringency index (NPI) and (**B**) the predicted (red solid line) and the forecasted (red dashed line) any dose COVID-19 vaccination coverage rates with 95% Bayesian credible intervals (shaded grey region) (pharmaceutical interventions). The black dashed lines show the beginning (1^st^ January 2022) and the end (31^st^ March 2022) of the forecast period. The red horizontal dot-dashed line indicates the 70% COVID-19 vaccination coverage required to achieve herd immunity [72].

**Fig. 4:**
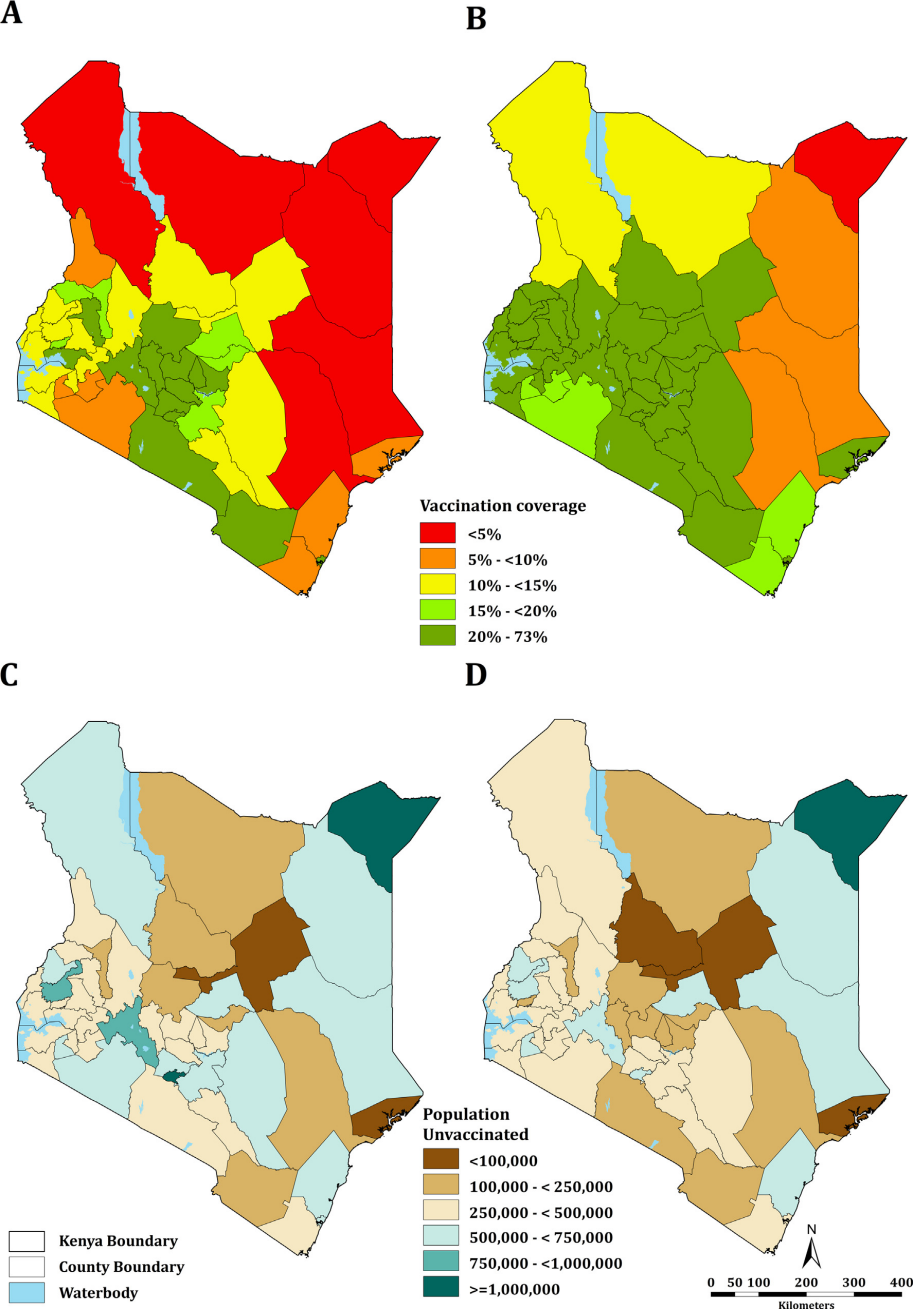
Modelled mean COVID-19 vaccination coverage at sub-national level; (**A**) before the forecast period and (**B**) at the end of the forecast period. The population unvaccinated (unmet need) (**C**) before forecast and (**D**) after forecast.

Fig. 3A shows the number of SARS CoV-2 cases superimposed with the COVID-19 stringency index which represents the NPIs employed. Fig. 3B shows the number of COVID-19 cases overlaid with the predicted any dose vaccination coverages. Nationally, the vaccination coverage rate rose from 1.90% (95% CI: 1.89–1.91) – approximately 497,510 people in April to 16.70% (95% CI: 16.66–16.74) – approximately 4.4 million people in December 2021. The vaccination coverage forecast, which began on 1^st^ January 2022, shows a continued increase in coverage from 17.56% (95% CI: 17.52–17.60) – approximately 4.6 million people to 30.75% (95% CI: 25.04–36.96) – approximately 8.1 million people at the end of the forecasting period (End of March 2022). The coverages at county level are shown in Fig. 4 with (A) showing the coverage before the forecast period and (B) at the end of the forecast period. At sub-national level, the vaccination coverage ranged from 1.51% (95% CI: 1.51–1.52) – approx. 17,000 people to 54.28% (95% CI: 54.24–54.32) – approx. 1.3 million people before forecast and 3.59% (95% CI: 2.62–4.83) – approx. 40,000 people to 73.75% (95% CI: 67.35–79.53) – approx. 1.8 million people after forecast periods, both in Mandera and Nairobi counties respectively (Table SI 2). As of December 2021, six counties had a vaccination coverage of less than 5%. These counties include Garissa, Mandera, Marsabit, Tana River, Turkana, and Wajir. 13 counties had a coverage of more than 20% in this period including Embu, Kajiado, Kirinyaga, Kisumu, Laikipia, Mombasa, Murang’a, Nairobi, Nakuru, Nyandarua, Nyeri, Taita Taveta and Uasin Gishu. At the end of the forecast period, only one county, Mandera, will have a vaccination coverage of less than 5% and 35 counties will have a coverage of more than 20%.

### 3.5 Unmet need for COVID-19 vaccination coverage

The difference in the number of people given the vaccine and the target population in the different counties suggests an unmet need in vaccination coverage. The population of people unvaccinated at county level is shown in Fig. 4 with (C) showing the numbers before the forecast period and (D) at the end of the forecast period. Nationally, approximately 21 million people are still unvaccinated as of December 2021 and, in the absence of accelerated vaccine uptake, over 17.2 million people may not be vaccinated by end March 2022. At the sub-national level, the population not vaccinated ranges from over 59,000 people in Lamu county to over 1.1 million people in Nairobi before forecast and over 51,000 people in Lamu to approximately 1.1 million people in Mandera county after forecast. For the 15 counties with the proportion of population within 1hr of a COVID-19 vaccination centre was below the national average (Fig. 2), the coverage ranged from 1.51% to 32.68% before the forecast period and from 3.59% to 53.88% at the end of the forecast.

## 4 Discussion

This study estimated physical accessibility and coverage of COVID-19 vaccinations at sub-national level using a statistical approach. Nationally, the average travel time to a designated COVID-19 vaccination site (*n* = 622) was 75.5 min (Range: 62.9–94.5 min) and over 87% of the population >18 years reside within 1 hr of a vaccination site. At national level, the COVID-19 vaccination coverage rate in December 2021 was 16.70% (95% CI: 16.66–16.74) – approx. 4.4 million but was lower in rural areas by 27.8%. Vaccination rate was higher amongst population >58 years by 37.6% (Table 1). Based on the current vaccination rate, it was estimated that the vaccination coverage amongst people >18 years is likely to increase to 30.75% (95% CI: 25.04–36.96) – approx. 8.1 million by end of March 2022. However, this is likely to vary at sub-national level with 12 counties not likely to achieve 20% coverage by March 2022 (Table SI 2). For urban counties (Nairobi and Mombasa), coverage estimates are likely to increase to 67.54% (approx. 2.1 million people) by end of March 2022. These results highlight sub-national level inequalities and are, thus, important in targeting and improving vaccination coverage in marginalized populations.

Increasing the number and availability of vaccination sites is one possible way of increasing vaccination coverage [17]. Other methods of increasing vaccination coverage include implementing school/institutional based vaccination programmes [73], [74], and adopting effective mandatory vaccinations among the priority groups. Counties in Kenya that would benefit from this strategy (increase in the number of vaccination sites) through permanent or mobile units include Garissa, Isiolo, Kilifi, Lamu, Mandera, Marsabit, Narok, Samburu, Tana River, Turkana, Wajir and West Pokot. These counties account for 19.8% of the population >18 years with average travel times to vaccination sites of 194.9 min. Seven counties (Mandera, Marsabit, Samburu, Tana River, Turkana, Wajir and West Pokot) had significant percentage of population residing more than 1 hr to a vaccination site ranging from 48.1% in West Pokot to 77.5% in Marsabit and translates to 67.29% of their population (Fig. 2). These mentioned counties have also been previously identified as marginalized through a 2014 report published by the Commission on Resource Allocation (CRA) [75]. The coverages in these marginalized counties are unlikely to reach 20% at the end of the forecast period except for Samburu, and the intervention measures and resource allocation should, therefore, be prioritized in these areas. However, efforts that focus on increasing supply alone are unlikely to be effective. Vaccine hesitancy remains a major barrier to COVID-19 vaccination uptake [5], [76], [77], [78]. Methods of reducing vaccine hesitancy include providing sensitization campaigns on the disease risk and importance of vaccinations through posters and media advertisements [74], [79].

Vaccination coverage in urban areas was higher by 27.8% as compared to rural areas. There are several reasons for higher coverages in urban areas, including but not limited to shorter travel times also reported in other studies [54], [55], increased availability of vaccines, higher literacy [23], [24], occupation [23], [24], [25], [26], and low hesitancy rates [5]. Despite the higher rates of vaccination coverage in urban areas, it is projected to reach 73.75% in Nairobi and 44.56% in Mombasa by March 2022. The high coverage rates in the urban counties is mainly attributed to the high density of vaccination sites portraying an ‘urban advantage’. However, the urban averages in coverage can mask inequalities within urban areas which are minimized after adjusting for wealth [80].

The methodology for estimating coverage rates at sub-national level used aggregated data from vaccination sites within the county. Data was not available from each vaccination site due to data protection reasons and confidentiality, and data governance. The issue that affect routine data in sub-Saharan Africa (SSA) for diseases [81] also impacts COVID-19 data. Fine-scale mapping of vaccination coverage could be explored once data is anonymized and is available for each vaccination site. Such approaches have been used for the estimation of childhood vaccination coverages such as for measles and DTP [27], [28], [82], [83]. Fine-scale mapping could refine geographical targeting to reach physically marginalized population. The effect of travel time to the nearest vaccination site was not assessed for changing road conditions in rural areas, effects of by-passing due to vaccine stock-outs [54], [55] and perceived quality of services offered by the vaccination site [84]. Previous studies that examined changing road conditions showed longer journeys during wet seasons as compared to drier seasons [62], [85]. This suggests that the computed travel times could underestimate travel times in rural areas with unpaved roads affected by changing road conditions. Empirical data to test these assumptions further were not available.

The present analysis identifies gaps in COVID-19 vaccination coverage at the population level amongst >18 years. Since Kenya recorded its first COVID-19 case in March 2020, several control measures have been implemented at an enormous cost to society. Vaccination is one key pharmaceutical intervention to combat the pandemic, and while there has been increasing vaccination coverage since March 2021, it is projected to reach 31% if the current rate is sustained. At sub-national level, a targeted strategy prioritizing geographically marginalized communities is necessary to achieve national targets for vaccination.

## 5 Ethics statement

The study involved the assembly of secondary data, previously published or part of national surveys. Ethical approvals for all survey data assembled was presumed sought by national investigators.

## CRediT authorship contribution statement

**Samuel K. Muchiri:** Conceptualization, Data curation, Methodology, Writing – original draft, Writing – review & editing. **Rose Muthee:** Writing – review & editing. **Hellen Kiarie:** Data curation, Writing – review & editing. **Joseph Sitienei:** Data curation, Writing – review & editing. **Ambrose Agweyu:** Supervision, Writing – review & editing. **Peter M. Atkinson:** Supervision, Writing – review & editing. **C. Edson Utazi:** Methodology, Supervision, Writing – review & editing. **Andrew J. Tatem:** Supervision, Writing – review & editing. **Victor A. Alegana:** Conceptualization, Data curation, Funding acquisition, Methodology, Supervision, Project administration, Writing – original draft, Writing – review & editing.

## Declaration of Competing Interest

All authors declare no competing interests.

## Data Availability

COVID-19 vaccination data are available with open access provided by the Ministry of Heath Kenya (https://www.health.go.ke/#1621663315215-d6245403-4901).

## References

[1] World Health Organization. WHO Director-General’s opening remarks at the media briefing on COVID-19-11 March 2020. Geneva, Switzerland; 2020.

[2] Ministry of Health. Ministry of Health; 2021.

[3] Coronavirus W. Dashboard| WHO Coronavirus (COVID-19) Dashboard With Vaccination Data; 2021.

[4] Barasa E., Kazungu J., Orangi S., Kabia E., Ogero M., Kasera K. (2021). Assessing the Indirect Health Effects of the COVID-19 Pandemic in Kenya. CGD Work Pap..

[5] Orangi S., Pinchoff J., Mwanga D., Abuya T., Hamaluba M., Warimwe G. (2021). Assessing the level and determinants of COVID-19 Vaccine Confidence in Kenya. medRxiv..

[6] Alvi M.M., Sivasankaran S., Singh M. (2020). Pharmacological and non-pharmacological efforts at prevention, mitigation, and treatment for COVID-19. J Drug Target.

[7] Bokharee N., Khan Y.H., Khokhar A., Mallhi T.H., Alotaibi N.H., Rasheed M. (2021). Pharmacological interventions for COVID-19: a systematic review of observational studies and clinical trials. Expert Rev Anti-infective Therapy.

[8] Chakraborty R., Parvez S. (2020). COVID-19: An overview of the current pharmacological interventions, vaccines, and clinical trials. Biochem Pharmacol.

[9] D'Souza R., Ashraf R., Rowe H., Zipursky J., Clarfield L., Maxwell C. (2021). Pregnancy and COVID-19: pharmacologic considerations. Ultrasound Obstet Gynecol.

[10] Khalili M., Chegeni M., Javadi S., Farokhnia M., Sharifi H., Karamouzian M. (2020). Therapeutic interventions for COVID-19: a living overview of reviews. Therapeutic Adv Respiratory Dis..

[11] Kim M.S., An M.H., Kim W.J., Hwang T.-H. (2020). Comparative efficacy and safety of pharmacological interventions for the treatment of COVID-19: A systematic review and network meta-analysis. PLoS Med.

[12] Ostuzzi G., Gastaldon C., Papola D., Fagiolini A., Dursun S., Taylor D. (2020). Pharmacological treatment of hyperactive delirium in people with COVID-19: rethinking conventional approaches. Therapeutic Adv Psychopharmacol..

[13] World Health Organization. Therapeutics and COVID-19: living guideline. In: Organization WH, editor.; 2022. 35917393

[14] World Health Organization. COVID-19 advice for the public: Getting vaccinated; 2021.

[15] Geldsetzer P., Reinmuth M., Ouma P.O., Lautenbach S., Okiro E.A., Bärnighausen T. (2020). Mapping physical access to health care for older adults in sub-Saharan Africa and implications for the COVID-19 response: A cross-sectional analysis. The Lancet Healthy Longevity..

[16] World Health Organization. Status of COVID-19 Vaccines within WHO EUL/PQ evaluation process; 2021.

[17] Ministry of Health. National COVID-19 Vaccine Deployment Plan; 2021.

[18] Deplanque D., Launay O. (2021). Efficacy of Covid-19 vaccines: from clinical trials to real life. Therapies..

[19] Institute of Health Metrics and Evaluation (IHME). COVID-19 vaccine efficacy summary; 2021.

[20] Centres for Disease Control and Prevention. Benefits of getting a COVID-19 vaccine.

[21] GAVI The vaccination alliance. COVAX; 2021.

[22] John Muchangi. Kenya on course to hit 10 million vaccination target by December. The Star; 2021.

[23] Elizabeth K., George K., Raphael N., Moses E. (2015). Factors influencing low immunization coverage among children between 12–23 months in East Pokot, Baringo Country, Kenya. Int J Vaccines..

[24] Galadima A.N., Zulkefli N.A.M., Said S.M., Ahmad N. (2021). Factors influencing childhood immunisation uptake in Africa: a systematic review. BMC Public Health..

[25] Legesse E., Dechasa W. (2015). An assessment of child immunization coverage and its determinants in Sinana District, Southeast Ethiopia. BMC Pediatrics..

[26] Al-Mohaithef M., Padhi B.K. (2020). Determinants of COVID-19 vaccine acceptance in Saudi Arabia: a web-based national survey. J Multidisciplinary Healthcare.

[27] Utazi C.E., Nilsen K., Pannell O., Dotse-Gborgbortsi W., Tatem A.J. (2021). District-level estimation of vaccination coverage: discrete vs continuous spatial models. Stat Med.

[28] Utazi C.E., Wagai J., Pannell O., Cutts F.T., Rhoda D.A., Ferrari M.J. (2020). Geospatial variation in measles vaccine coverage through routine and campaign strategies in Nigeria: Analysis of recent household surveys. Vaccine..

[29] Utazi C., Thorley J., Alegana V., Ferrari M., Nilsen K., Takahashi S. (2019). A spatial regression model for the disaggregation of areal unit based data to high-resolution grids with application to vaccination coverage mapping. Stat Methods Med Res.

[30] Collaborators LBoDVC (2021). Mapping routine measles vaccination in low-and middle-income countries. Nature.

[31] Utazi C.E., Thorley J., Alegana V.A., Ferrari M.J., Takahashi S., Metcalf C.J.E. (2019). Mapping vaccination coverage to explore the effects of delivery mechanisms and inform vaccination strategies. Nat Commun.

[32] Mosser J.F., Gagne-Maynard W., Rao P.C., Osgood-Zimmerman A., Fullman N., Graetz N. (2019). Mapping diphtheria-pertussis-tetanus vaccine coverage in Africa, 2000–2016: a spatial and temporal modelling study. The Lancet..

[33] Pfeffermann D. (2013). New important developments in small area estimation. Statistical Sci..

[34] Rao J.N., Molina I. (2015).

[35] Wakefield J, Okonek T, Pedersen J. Small Area Estimation of Health Outcomes. arXiv preprint arXiv:200610266; 2020.

[36] Tatem A.J. (2017). WorldPop, open data for spatial demography. Sci Data.

[37] Besag J., Kooperberg C. (1995). On conditional and intrinsic autoregressions. Biometrika.

[38] Held L, Rue H. Conditional and intrinsic autoregressions. Handbook of spatial statistics; 2010. p. 201–16.

[39] Banerjee S., Carlin B.P., Gelfand A.E. (2003).

[40] Alegana V.A., Atkinson P.M., Wright J.A., Kamwi R., Uusiku P., Katokele S. (2013). Estimation of malaria incidence in northern Namibia in 2009 using Bayesian conditional-autoregressive spatial–temporal models. Spatial Spatio-temporal Epidemiol.

[41] Moraga P. (2018). Small Area Disease Risk Estimation and Visualization Using R. R J..

[42] Magdalene Saya. Kenya to prioritise Covid-19 vaccination for those aged 58 years and above. The Star; 2021.

[43] Kyobutungi C. (2021). The ins and outs of Kenya’s COVID-19 vaccine rollout plan. The Conversation.

[44] Our world in Data. Our World in Data; 2021.

[45] Hale T., Angrist N., Goldszmidt R., Kira B., Petherick A., Phillips T. (2021). A global panel database of pandemic policies (Oxford COVID-19 Government Response Tracker). Nat Hum Behav.

[46] Maina J., Ouma P.O., Macharia P.M., Alegana V.A., Mitto B., Fall I.S. (2019). A spatial database of health facilities managed by the public health sector in sub Saharan Africa. Sci Data.

[47] WorldPop S. of G. and ESU of, Department of Geography and Geosciences, U. of L., Département de Géographie, U. de N. & Center for International Earth Science Information Network (CIESIN), CU; 2018.

[48] Macrotrends. Kenya Population Growth Rate 1950-2021.

[49] Stevens F.R., Gaughan A.E., Linard C., Tatem A.J. (2015). Disaggregating census data for population mapping using random forests with remotely-sensed and ancillary data. PLoS ONE.

[50] Breiman L. (2001). Random forests. Mach Learn.

[51] Bondarenko M, Nieves J, Stevens F, Gaughan A, Tatem A, Sorichetta A. wpgpRFPMS: Random Forests population modelling R scripts, version 0.1. 0. Southampton, UK: University of Southampton; 2020.

[52] Kenya National Bureau of Statistics, Ministry of Health/Kenya, National AIDS Control Council/Kenya, Kenya Medical Research Institute, Population NCf, Development/Kenya. Kenya Demographic and Health Survey 2014. Rockville, MD, USA; 2015.

[53] The DHS Program.

[54] Joseph N.K., Macharia P.M., Ouma P.O., Mumo J., Jalang’o R., Wagacha P.W. (2020). Spatial access inequities and childhood immunisation uptake in Kenya. BMC Public Health..

[55] Macharia P.M., Mumo E., Okiro E.A. (2021). Modelling geographical accessibility to urban centres in Kenya in 2019. PLoS ONE.

[56] UNEP-WCMC. The World database on protected areas; 2017.

[57] Kenya Wildlife Service. Overview of national parks and reserves; 2021.

[58] Bingham Heather C, Lewis Edward, Stewart Jessica, Juffe-Bignoli Diego, MacSharry Brian, Amy Milam NK. User Manual for the World Database on Protected Areas and world database on other effective area based conservation measures: 1.6; 2019.

[59] Regional Centre for Mapping of Resources for Development. RCMRD geoportal.

[60] Macharia P.M., Ouma P.O., Gogo E.G., Snow R.W., Noor A.M. (2017). Spatial accessibility to basic public health services in South Sudan. Geospatial Health.

[61] Noor A.M., Amin A.A., Gething P.W., Atkinson P.M., Hay S.I., Snow R.W. (2006). Modelling distances travelled to government health services in Kenya. Trop Med Int Health.

[62] Ouma P., Macharia P.M., Okiro E., Alegana V. (2021). Methods of Measuring Spatial Accessibility to Health Care in Uganda. Practicing Health Geogr: African Context.

[63] Ray N., Ebener S. (2008). AccessMod 3.0: computing geographic coverage and accessibility to health care services using anisotropic movement of patients. Int J Health Geographics.

[64] Tobler W. Three presentations on geographical analysis and modelling. Santa Barbara, CA 93106-4060: National Center for Geographic Information and Analysis, University of California; 1993.

[65] Rue H., Martino S., Chopin N. (2009). Approximate Bayesian inference for latent Gaussian models by using integrated nested Laplace approximations. J Royal Stat Soc: Series b (Stat Methodol)..

[66] Martins T.G., Simpson D., Lindgren F., Rue H. (2013). Bayesian computing with INLA: new features. Comput Stat Data Anal.

[67] Fuglstad G.-A., Simpson D., Lindgren F., Rue H. (2019). Constructing priors that penalize the complexity of Gaussian random fields. J Am Stat Assoc.

[68] Blangiardo M., Cameletti M., Baio G., Hv R. (2013). Spatial and spatio-temporal models with R-INLA. Spat Spatio-temporal Epidemiol..

[69] Czado C., Gneiting T., Held L. (2009). Predictive model assessment for count data. Biometrics..

[70] Held L, Schrödle B, Rue H. Posterior and cross-validatory predictive checks: a comparison of MCMC and INLA. Statistical modelling and regression structures: Springer; 2010. p. 91–110.

[71] Ministry of Devolution and the ASALS.

[72] Aschwanden C. (2021). Five reasons why COVID herd immunity is probably impossible. Nature.

[73] Ozawa S., Yemeke T.T., Thompson K.M. (2018). Systematic review of the incremental costs of interventions that increase immunization coverage. Vaccine..

[74] CDC. Examples of Evidence-Based Solutions to Increase Vaccine Confidence and Uptake.

[75] Commission on Revenue Allocation (CRA). Policy on the criteria for identifying marginalised areas and sharing of the equalisation fund; 2014.

[76] Dror A.A., Eisenbach N., Taiber S., Morozov N.G., Mizrachi M., Zigron A. (2020). Vaccine hesitancy: the next challenge in the fight against COVID-19. Eur J Epidemiol.

[77] Dzinamarira T., Nachipo B., Phiri B., Musuka G. (2021). COVID-19 vaccine roll-out in South Africa and Zimbabwe: urgent need to address community preparedness, fears and hesitancy. Vaccines.

[78] Lazarus J.V., Ratzan S.C., Palayew A., Gostin L.O., Larson H.J., Rabin K. (2021). A global survey of potential acceptance of a COVID-19 vaccine. Nat Med.

[79] Acampora A., Grossi A., Barbara A., Colamesta V., Causio F.A., Calabrò G.E. (2020). Increasing HPV vaccination uptake among adolescents: A systematic review. Int J Environ Res Public Health.

[80] UNICEF. Advantage or paradox? The challenge for children and young people of growing up urban: United Nations; 2019.

[81] Alegana V.A., Okiro E.A., Snow R.W. (2020). Routine data for malaria morbidity estimation in Africa: challenges and prospects. BMC Med.

[82] Utazi C.E., Thorley J., Alegana V.A., Ferrari M.J., Takahashi S., Metcalf C.J.E. (2018). High resolution age-structured mapping of childhood vaccination coverage in low and middle income countries. Vaccine..

[83] Takahashi S., Metcalf C.J.E., Ferrari M.J., Tatem A.J., Lessler J. (2017). The geography of measles vaccination in the African Great Lakes region. Nat Commun.

[84] Alford-Teaster J., Lange J.M., Hubbard R.A., Lee C.I., Haas J.S., Shi X. (2016). Is the closest facility the one actually used? An assessment of travel time estimation based on mammography facilities. Int J Health Geographics.

[85] Blanford J.I., Kumar S., Luo W., MacEachren A.M. (2012). It’sa long, long walk: accessibility to hospitals, maternity and integrated health centers in Niger. Int J Health Geographics.

