# Supplementary material for: Unmet need for COVID-19 vaccination coverage in Kenya
Source: Vaccine. 2022 Mar 18;40(13):2011–9. doi: 10.1016/j.vaccine.2022.02.035 (PMC8841160; doi:10.1016/j.vaccine.2022.02.035)
Supplement: Supplementary Data 1 [file mmc1.docx]

**Unmet need for COVID-19 vaccination coverage in Kenya**

**Samuel K. Muchiri ^a^*, Rose Muthee ^b^, Hellen Kiarie ^b^, Joseph Sitienei ^b^, Ambrose Agweyu ^c^, Peter M. Atkinson ^d, e, f^, C. Edson Utazi ^f, g^, Andrew J. Tatem ^g^, Victor A. Alegana ^a, e^**

1. Population Health Unit, Kenya Medical Research Institute-Wellcome Trust Research Programme, Nairobi, Kenya.
2. Department of Health Informatics, Monitoring and Evaluation, Ministry of Health, Nairobi, Kenya.
3. Epidemiology and Demography, KEMRI-Wellcome Trust Research Programme Nairobi, Kenya
4. Lancaster Environment Centre, Lancaster University, Lancaster, LA1 4YQ, UK
5. Geography and Environmental Science, University of Southampton, Highfield, Southampton, SO17 1BJ, UK
6. Institute of Geographic Sciences and Natural Resource Research, Chinese Academy of Sciences, Beijing, 100101, China
7. WorldPop, School of Geography and Environmental Science, University of Southampton, Southampton, UK
8. Southampton Statistical Sciences Research Institute, University of Southampton, Southampton, UK

**Supplementary Information**

**Table of Contents**

[**1** **Estimating population proportions for age >18 years** 2](#_Toc83290523)

[*1.1* *Modelling age proportions using household survey data* 3](#_Toc83290524)

[*1.2* *Prediction of age proportions at fine spatial resolution* 4](#_Toc83290525)

[**2** **Model Fitting results and validation** 5](#_Toc83290526)

[**3** **COVID-19 vaccination metrics** 6](#_Toc83290527)

[**4** **References** 8](#_Toc83290528)

# **Estimating population proportions for age >18 years**

## *Modelling age proportions using household survey data*

The age composition in Kenya was modelled based latest DHS [1]. The prediction of age proportions spatially required exploiting both the spatial covariance structure in the DHS cluster data and the relationships with covariates. Here, only urban or rural was the only considered covariate although other covariates can be used in modelling age spatially. This urban and rural covariate was obtained from the global rural and urban mapping (GRUMP) project [2].

A Gamma distribution was used to define age proportions. Specifically, the individual level variable of age was assumed to follow a Gamma distribution with two parameters (shape and scale) defined at each DHS cluster. At the first stage, the two parameters were modelled in a bivariate linear regression model adjusting for urban or rural differences. At the second stage, a stationary Gaussian process $S$ was used to account for spatially correlated random variation.

Let $A_{ij}$be the age of the j^th^ individual from the DHS household census out of $m_{i}$at the location $x_{i}$ out of $n$. A conditional bivariate spatial stochastic process$(\alpha(x_{i}), \lambda(x_{i}))$ was used with $A_{ij}$ assumed to follow a Gamma distribution with mean $\alpha(x_{i})\lambda(x_{i})$ and variance$\alpha(x_{i})\lambda^{2}(x_{i})$. Here, $\alpha_{i}$and $\lambda_{i}$ is used as a shorthand notation for $\alpha(x_{i})$ and $\lambda(x_{i})$, respectively. A two-stage fitting procedure was used whereby in the first stage $\alpha_{i}$and $\lambda_{i}$ were estimated by maximizing the likelihood function of a Gamma distribution at each of the cluster locations$x_{i}$, given by:

$$L_{i}=\prod_{j=1}^{m_{i}} \frac{1}{\Gamma(\alpha_{i})\lambda_{i}^{\alpha_{i}}}A_{ij}^{\alpha_{i}-1}exp\left( -A_{ij}/\lambda_{i} \right)$$

and thus yielding $\hat{\alpha}(x_{i})$ and $\hat{\lambda}(x_{i})$ for $i=1,...n$. To spatially interpolate the estimates of the shape and scale parameters of the Gamma distribution at a cluster-level, a bivariate geostatistical model was used of the form:

$$log\left( \hat{\lambda}(x_{i}) \right)=\beta_{0}+\beta_{1}d(x_{i})+S(x_{i})+U_{i}$$

$$log\left( \hat{\alpha}(x_{i}) \right)=\gamma_{0}+\gamma_{1}d(x_{i})+\delta S(x_{i})+V_{i}$$

Where $S(x_{i})$ is a stationary and isotropic Gaussian process with mean zero, variance $\sigma^{2}$and exponential correlation function with scale parameter $\phi$. The regression parameter $\delta$ regulates the linear association between the log-transformed scale and shape parameters of the Gamma distribution. The pair of random variables $(U_{i}, V_{i})$is used to account for the sampling variability of the maximum likelihood estimators $(\hat{\alpha}(x_{i}), \hat{\lambda}(x_{i}))$, hence this was modelled as a set of independent bivariate Gaussian distributions with mean zero and variances $\tau_{1}^{2}$ and $\tau_{2}^{2}$, respectively.Model specification was completed by letting $Y^{\top}=\log(\hat{\alpha}\left( x_{i} \right), \ldots\hat{\alpha}\left( x_{n} \right), \hat{\lambda}\left( x_{i} \right), \ldots, \hat{\lambda}(x_{n}))$.

Then $Y^{\top}$ is a multivariate Gaussian distribution:

$$\left( \begin{matrix} \log(\hat{\lambda}) \\ \log(\hat{\alpha}) \end{matrix} \right)\sim MVN \left( \mu=\left( \begin{matrix} D\beta\\ D\gamma\end{matrix} \right), \Omega=\left( \begin{matrix} \sigma^{2}\Sigma+\tau_{1}^{2}B_{1} & \delta\sigma^{2}\Sigma+\sqrt{\tau_{1}^{2}\tau_{2}^{2}}C \\ \delta\sigma^{2}\Sigma+\sqrt{\tau_{1}^{2}\tau_{2}^{2}}C & \sigma^{2}\Sigma+\tau_{2}^{2}B_{2} \end{matrix} \right) \right)$$

where $D$ is a design matrix of covariates $\beta^{\top}= (\beta_{0},\beta_{1})$; $\gamma^{\top}= (\gamma_{0},\gamma_{1})$; $\Sigma_{\mathrm{ij}}= exp({-u}_{\mathrm{ij}}/\phi)$, $u_{\mathrm{ij}}$ is a Euclidian distance between $x_{i}$ and $x_{j}$ and $B_{1}, B_{2},C$ are diagonal matrices that adjust for the variances of $\hat{\alpha}\left( x_{i} \right)$ and $\hat{\lambda}\left( x_{j} \right)$ and the covariance between $\hat{\alpha}\left( x_{i} \right)$ and $\hat{\lambda}\left( x_{j} \right)$, respectively, given by the observed Fisher information obtained from the likelihood:

$$B_{1}=\left( \begin{matrix} Var\left( \hat{\alpha}\left( x_{i} \right) \right), if i=j \\ 0, otherwise \end{matrix} \right)$$

$$B_{2}=\left( \begin{matrix} Var\left( \hat{\lambda}\left( x_{j} \right) \right), if i=j \\ 0, otherwise \end{matrix} \right)$$

$$C=\left( \begin{matrix} Cov\left( \hat{\alpha}\left( x_{i} \right), \hat{\lambda}\left( x_{j} \right) \right), if i=j \\ 0, otherwise \end{matrix} \right)$$

The log-likelihood of $\theta^{\top}=(\beta_{0}, \beta_{1}, \gamma_{0}, \gamma_{1}, \delta, \sigma^{2}, \phi,\tau_{1},\tau_{2})$ is then given by

q$L\left( \theta\right)= -0.5 \left( \log\left| \Omega\right|+\left( y-\mu\right)^{\top}\Omega\left( y-\mu\right) \right)$

## *Prediction of age proportions at fine spatial resolution*

The first objective was to predict the pair of Gamma distribution parameters $\left( {\hat{\lambda}(x}_{i}^{*}), {\alpha(x}_{i}^{*}) \right)$ at each prediction location. Therefore, a predictive target distribution was defined as $T^{\top}=\left( {T(x}_{1}^{*}), \ldots{T(x}_{n}^{*}) \right)$. The conditional distribution of $T$ given the data $Y=y$ and urban/rural covariate at each of the prediction locations $x_{i}^{*}$ is a multivariate Gaussian with mean:

$$\mu^{*}+ P\Omega^{-1}(y-\mu)$$

where $\mu^{*}= {((D}^{*}\beta)^{\top}, {(D}^{*}\gamma)^{\top})^{\top}$ and $D^{*}$ is the design matrix of explanatory variables at the prediction locations, $P$ is the cross-covariance matrix and $\beta$ and $\gamma$ are the maximum likelihood estimates of a vector of the regression coefficients reported in equation 3.

To predict the proportion of the population belonging to a specified age class $a_{0}-a_{1}$ at $x^{*}=\left( x_{1}^{*}\ldots x_{q}^{*} \right)$, 10,000 samples were drawn from the predictive target distribution. For each of the 10,000 draws of $\left( {\hat{\lambda}(x}_{i}^{*}), {\alpha(x}_{i}^{*}) \right)$ the probability of class membership $a_{0}-a_{1}$ was computed $F\left( a_{1}; {\hat{\lambda}(x}_{i}^{*} \right), {\alpha(x}_{i}^{*}))- F\left( a_{0}; {\hat{\lambda}(x}_{i}^{*} \right), {\alpha(x}_{i}^{*}))$with $F(\cdot)$ as the cumulative distribution function of the Gamma distribution. This were summarise using the mean and 95% predictive intervals.

# **Model Fitting results and validation**

**Table SI 1:** Comparison of the Bayesian temporal models. Model 1 with no spatial random effect and Model 2 with spatial random effects and validation metrics.

|  |  | | | | | |
| --- | --- | --- | --- | --- | --- | --- |
| Model | DIC | **WAIC** | **Marginal likelihood** | **R^2^** | **RMSE** | **MAE** |
| Model 1 | 9,350,836.35 | 1,582,710.43 | -2,702,019.05 | - | - | - |
| Model 2 | 1,295,249.80 | 176,059.76 | -102,890.07 | 0.965 | 1.123 | 0.700 |


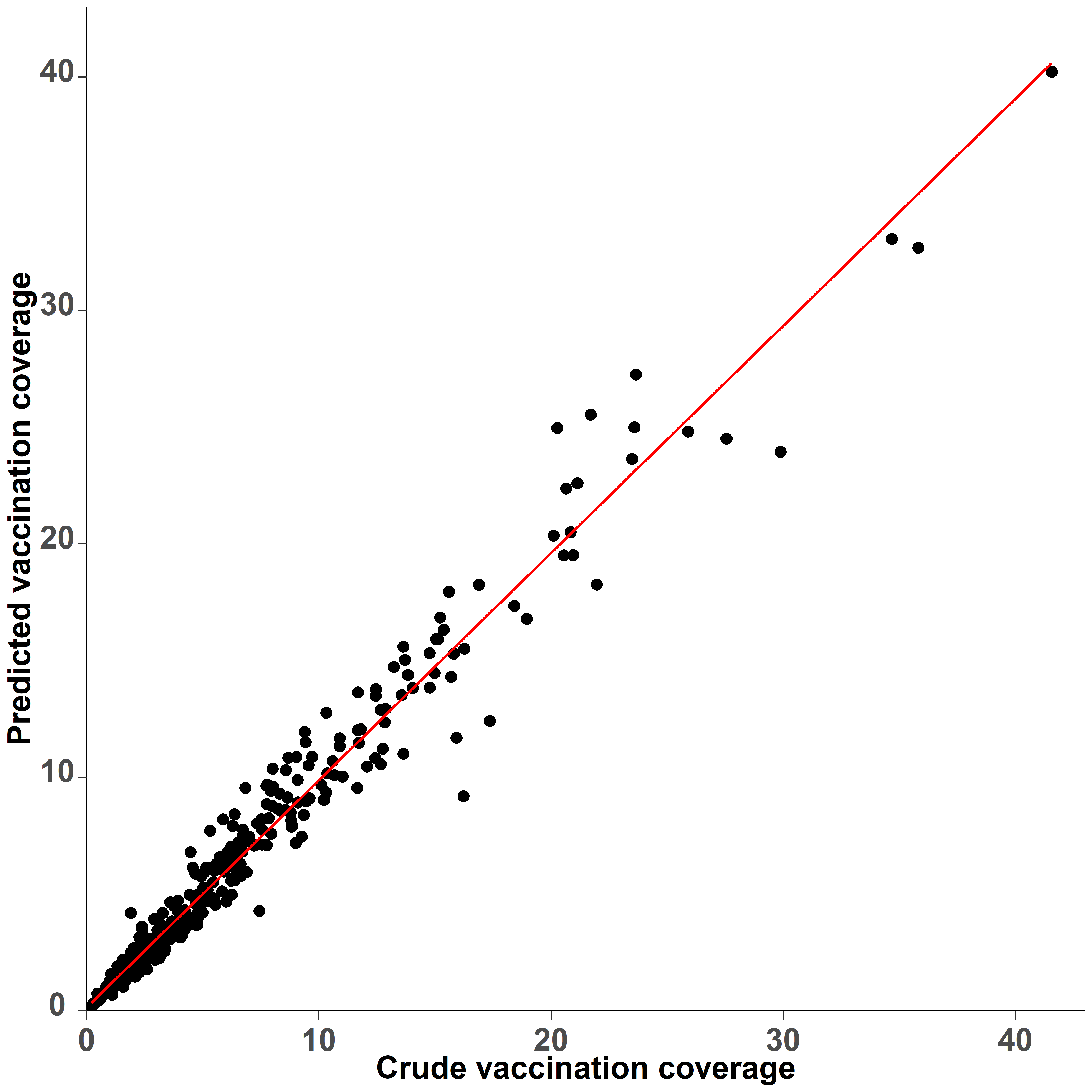


**Figure SI 1:** Validation scatter plot showing the association between the observed and the predicted COVID-19 vaccination coverages (n = 369).

# **COVID-19 vaccination metrics**

**Table SI 2:** Summary of COVID-19 vaccination sites, modelled travel times to the vaccination sites, population >18yrs and vaccination coverages at sub-national level.

| **County** | **Number of vaccination sites** | **Mean travel time in minutes to vaccination site**  **(± 20% mean speed variation)** | **Population >18years 2021** | **Any dose vaccination coverage before forecast (95% CI)** | **Any dose vaccination coverage after forecast**  **(95% CI)** |
| --- | --- | --- | --- | --- | --- |
| Baringo | 9 | 63.7 (53.0 -79.7) | 366,265 | 12.11 (12.07 – 12.14) | 25.09 (19.56 – 31.38) |
| Bomet | 1 | 27.2 (22.6 – 34.1) | 499,048 | 8.69 (8.67 – 8.72) | 18.93 (14.45 – 24.18) |
| Bungoma | 20 | 12.6 (10.4 – 15.8) | 794,071 | 12.40 (12.37 – 12.44) | 25.55 (19.94 – 31.93) |
| Busia | 8 | 20.8 (17.3 -26.1) | 437,260 | 12.21 (12.18 – 12.24) | 25.30 (19.72 – 31.64) |
| Elgeyo-Marakwet | 1 | 62.3 (51.8 – 77.9) | 256,004 | 15.18 (15.14 – 15.23) | 30.29 (23.99 – 37.29) |
| Embu | 6 | 31.7 (26.3 – 39.8) | 356,745 | 24.55 (24.50 – 24.60) | 44.21 (36.60 – 52.12) |
| Garissa | 12 | 217.7 (181.3 – 272.3) | 687,340 | 2.71 (2.69 – 2.72) | 6.31 (4.64 – 8.40) |
| Homa Bay | 20 | 22.7 (18.8 – 28.4) | 526,778 | 12.76 (12.73 – 12.79) | 26.17 (20.45 – 32.65) |
| Isiolo | 3 | 176.0 (146.6 – 220.1) | 81,665 | 13.71 (13.64 – 13.78) | 27.79 (21.83 – 34.49) |
| Kajiado | 5 | 137.3 (114.4 – 171.8) | 447,507 | 28.85 (28.80 – 28.91) | 49.44 (41.64 – 57.36) |
| Kakamega | 12 | 13.8 (11.5 -17.4) | 1,034,908 | 14.95 (14.92 – 14.98) | 28.69 (22.58 – 35.53) |
| Kericho | 16 | 21.8 (18.1 – 27.3) | 535,732 | 14.32 (14.28 – 14.35) | 28.82 (22.73 – 35.63) |
| Kiambu | 54 | 15.0 (12.4 -18.8) | 1,021,086 | 34.48 (34.44 – 34.53) | 54.39 (46.57 – 62.14) |
| Kilifi | 13 | 96.7 (80.5 – 121.0) | 670,861 | 7.72 (7.70 – 7.75) | 16.90 (12.82 – 21.73) |
| Kirinyaga | 8 | 12.5 (10.3 -15.7) | 360,315 | 23.61 (23.56 – 23.66) | 42.70 (39.69 – 55.41) |
| Kisii | 33 | 9.9 (8.2 – 12.5) | 689,306 | 9.32 (9.29 – 9.34) | 19.96 (15.29 – 25.41) |
| Kisumu | 34 | 14.5 (12.1 - 18.3) | 582,751 | 27.25 (27.20 – 27.30) | 47.45 (39.69 – 55.41) |
| Kitui | 20 | 83.0 (69.1 – 103.9) | 584,021 | 11.23 (11.20 – 11.26) | 23.63 (18.30 – 29.74) |
| Kwale | 7 | 76.5 (63.6 – 95.7) | 391,505 | 8.40 (8.37 – 8.43) | 18.11 (13.79 – 23.20) |
| Laikipia | 2 | 106.9 (89.0 – 133.7) | 262,477 | 32.68 (32.61 – 32.75) | 53.88 (46.03 – 61.69) |
| Lamu | 3 | 176.2 (146.8 – 220.4) | 65,483 | 9.81 (9.74 – 9.87) | 20.79 (15.96 – 26.40) |
| Machakos | 9 | 31.8 (26.4 – 39.9) | 723,467 | 19.72 (19.68 – 19.75) | 36.47 (29.43 – 44.07) |
| Makueni | 19 | 38.3 (31.8 – 48.0) | 560,756 | 11.10 (11.08 – 11.13) | 23.08 (17.84 – 29.11) |
| Mandera | 7 | 174.1 (145.0 – 217.8) | 1,122,484 | 1.51 (1.51 – 1.52) | 3.59 (2.62 – 4.83) |
| Marsabit | 7 | 294.0 (244.9 – 367.7) | 164,574 | 4.58 (4.55 – 4.61) | 10.41 (7.75 – 13.65) |
| Meru | 19 | 34.6 (28.8 – 43.4) | 875,166 | 15.31 (15.28 – 15.35) | 30.45 (24.13 – 37.46) |
| Migori | 30 | 20.1 (16.7 – 25.2) | 491,908 | 11.58 (11.55 – 11.61) | 24.17 (18.78 – 30.34) |
| Mombasa | 9 | 15.4 (12.7 – 19.4) | 671,180 | 24.99 (24.94 – 25.04) | 44.56 (36.95 – 52.47) |
| Murang’a | 12 | 16.0 (13.2 – 20.1) | 630,530 | 23.57 (23.53 – 23.61) | 42.69 (35.14 – 50.60) |
| Nairobi | 64 | 5.9 (4.8 – 7.4) | 2,482,138 | 54.28 (54.24 – 54.32) | 73.75 (67.35 – 79.53) |
| Nakuru | 34 | 30.1 (25.0 – 37.7) | 1,152,306 | 27.42 (27.39 – 27.47) | 48.61 (40.80 – 56.57) |
| Nandi | 7 | 23.7 (19.7 -29.8) | 540,054 | 14.91 (14.87 – 14.94) | 30.21 (23.92 – 37.19) |
| Narok | 9 | 105.8 (88.1 – 132.4) | 548,529 | 7.21 (7.18 – 7.24) | 15.93 (12.06 – 20.53) |
| Nyamira | 13 | 12.0 (10.0 -15.2) | 373,893 | 14.73 (14.69 – 14.77) | 29.50 (23.31 – 36.39) |
| Nyandarua | 5 | 25.0 (20.8 -31.4) | 374,558 | 27.26 (27.20 – 27.31) | 47.86 (40.10 – 55.78) |
| Nyeri | 3 | 28.6 (23.7 – 35.8) | 460,211 | 42.00 (41.95 – 42.06) | 63.36 (55.88 – 70.45) |
| Samburu | 1 | 219.8 (183.0 – 274.8) | 136,942 | 13.38 (13.32 – 13.43) | 27.27 (21.41 – 33.87) |
| Siaya | 19 | 19.9 (16.5 – 24.9) | 518,341 | 13.02 (12.99 – 13.05) | 26.55 (20.76 – 33.10) |
| Taita Taveta | 6 | 81.9 (68.2 – 102.5) | 198,578 | 21.76 (21.70 – 21.82) | 40.11 (32.78 – 47.89) |
| Tana River | 3 | 191.0 (159.1 – 238.9) | 170,272 | 3.94 (3.91 – 3.97) | 9.08 (6.74 – 11.95) |
| Tharaka-Nithi | 8 | 42.2 (35.1 – 52.9) | 230,678 | 17.87 (17.82 – 17.92) | 34.56 (27.72 – 42.10) |
| Trans Nzoia | 8 | 25.1 (20.8 – 31.4) | 556,047 | 17.53 (17.49 – 17.57) | 34.14 (27.38 – 41.51) |
| Turkana | 14 | 290.8 (242.2 – 363.6) | 530,932 | 4.76 (4.74 – 4.78) | 10.85 (8.09 – 14.22) |
| Uasin Gishu | 12 | 19.4 (16.1 – 24.4) | 652,951 | 29.54 (29.49 – 29.59) | 50.85 (43.04 – 58.73) |
| Vihiga | 6 | 9.9 (8.1 – 12.4) | 352,067 | 17.54 (17.49 – 17.58) | 34.05 (27.27 – 41.45) |
| Wajir | 6 | 254.3 (211.9 – 318.0) | 699,502 | 2.29 (2.28 – 2.30) | 5.39 (3.96 – 7.19) |
| West Pokot | 5 | 141.9 (118.2 – 177.5) | 315,599 | 6.04 (6.01 – 6.06) | 13.51 (10.16 – 17.54) |
| **Total** | **622** | **75.5 (62.9 – 94.5)** | **26,184,781** | **16.70 (16.66 – 16.74)** | **30.75 (25.04 – 36.96)** |

# **References**

[1] Kenya National Bureau of Statistics, Ministry of Health/Kenya, National AIDS Control Council/Kenya, Kenya Medical Research Institute, Population NCf, Development/Kenya. Kenya Demographic and Health Survey 2014. Rockville, MD, USA2015.

[2] Center for International Earth Science Information Network (CIESIN). Global Rural-Urban Mapping Project (GRUMP): Urban Extents. New York: CIESIN, Columbia University; 2004.
